# Connection of European particle therapy centers and generation of a common particle database system within the European ULICE-framework

**DOI:** 10.1186/1748-717X-7-115

**Published:** 2012-07-24

**Authors:** Kerstin A Kessel, Nina Bougatf, Christian Bohn, Daniel Habermehl, Dieter Oetzel, Rolf Bendl, Uwe Engelmann, Roberto Orecchia, Piero Fossati, Richard Pötter, Manjit Dosanjh, Jürgen Debus, Stephanie E Combs

**Affiliations:** 1 Department of Radiation Oncology, Heidelberg University Hospital, Im Neuenheimer Feld 400, 69120, Heidelberg, Germany; 2 CHILI GmbH, Friedrich-Ebert-Str. 2, 69221, Dossenheim, Germany; 3 Department of Medical Informatics, Heilbronn University, Max-Planck-Str. 39, 74081, Heilbronn, Germany; 4 National Centre for Oncological Hadrontherapy, via Caminadella 16, 20123, Milano, Italy; 5 Medical University of Vienna, Department of Radiotherapy, 18-20 Währinger Gürtel, 1090, Vienna, Austria; 6 CERN, 1211, Geneva 23, Switzerland

**Keywords:** Particle therapy, Multicenter clinical studies, Documentation system

## Abstract

**Background:**

To establish a common database on particle therapy for the evaluation of clinical studies integrating a large variety of voluminous datasets, different documentation styles, and various information systems, especially in the field of radiation oncology.

**Methods:**

We developed a web-based documentation system for transnational and multicenter clinical studies in particle therapy. 560 patients have been treated from November 2009 to September 2011. Protons, carbon ions or a combination of both, as well as a combination with photons were applied. To date, 12 studies have been initiated and more are in preparation.

**Results:**

It is possible to immediately access all patient information and exchange, store, process, and visualize text data, any DICOM images and multimedia data. Accessing the system and submitting clinical data is possible for internal and external users. Integrated into the hospital environment, data is imported both manually and automatically. Security and privacy protection as well as data validation and verification are ensured. Studies can be designed to fit individual needs.

**Conclusions:**

The described database provides a basis for documentation of large patient groups with specific and specialized questions to be answered. Having recently begun electronic documentation, it has become apparent that the benefits lie in the user-friendly and timely workflow for documentation. The ultimate goal is a simplification of research work, better study analyses quality and eventually, the improvement of treatment concepts by evaluating the effectiveness of particle therapy.

## Introduction

Particle therapy as an innovative and relatively new technique is of increasing interest in radiation oncology. Compared to standard radiation therapy (RT) with photons, the main advantages lie in the distinct physical characteristics of particles enabling a more precise dose delivery to the target and thereby sparing of normal tissue and organs at risk [1,2]. Heavier ions, such as carbon ions, additionally offer distinct biological characteristics leading to an increase in relative biological effectiveness (RBE) [3]: for example, it has been shown, that for glioblastoma cell lines, the RBE lies between 3 and 5 depending on cell type and endpoint [4].

For several further indications, clinical results of particle therapy have been shown to be beneficial [5,6]. Particle therapy, however, has been available only in a limited number of institutions. While proton therapy is more widespread, especially in the United States, carbon ion radiotherapy was only available in Japan and Germany. Beginning in 1997, treatment was performed at the Gesellschaft für Schwerionenforschung (GSI) in Darmstadt, Germany and since November 2009, treatment has become available within clinical routine at the Heidelberg Ion-Beam Therapy Center (HIT) in Heidelberg, Germany. In the near future, other European Centers will take up clinical operation. Recently, the CNAO (National Centre for Oncological Hadrontherapy) in Pavia, Italy, started patient treatment with particle therapy.

Since several European particle therapy initiatives are underway, and since clinical data, especially with respect to randomized clinical studies, still remains scarce [7], the logical consequence is to combine all efforts in the field of particle therapy and to generate a common platform for all patients treated with particle beams. Therefore, within the transnational access (TNA) pillar of the ULICE project (Union of Light Ions Centers in Europe) funded by the European Commission, the generation of a common database has been a main focus.

Researchers at the HIT started developing this common database and documentation system to conduct international, multicenter clinical studies where all patient data is gathered by the participating institutions. During this process, increasingly large amounts of patient data must be analyzed. In general, analyzing clinical studies, in particular retrospectively, which contain large patient groups, is rather difficult, because of size and heterogeneity of the data and the different documentation style within different departments. Especially radiation oncology as an interdisciplinary field must deal with a large variety of voluminous datasets from various information systems. This demands special coordination in data management. Therefore, we primarily need a documentation and management system integrated in the clinical environment, but preferably an additional, built-in possibility for immediate analysis of the collected data.

Nowadays, in the age of modern technology, the first choice exists in using Internet technologies for transnational access. It is easiest and most common to work together by using the web. It has not only the advantage of having a high user acceptance and intuitive usability, but also to be platform-independent. Especially in healthcare, it is crucial to have all patient information on hand - even on mobile devices [8] - particularly in radiotherapy where one is always involved with imaging information.

In this paper, we describe our approach and first steps to achieving an international web-based documentation system in particle therapy. Our main aim is to transfer results and experiences with treatment concepts and ideas to potential new ion centers about to be set up worldwide.

## Methods

### General database concept

The database is constructed to assemble clinical, biological, and physical data of all patients treated with particle therapy within the ULICE framework. To validate and establish the workflow in the database system, at first patients treated at the leading TNA institution HIT, have been included.

From November 2009 to September 2011 560 patients received treatment at the HIT. Protons (H1), carbon ions (C12) or a combination of both, as well as a combination with photons were applied to the target with the intensity-modulated rasterscanning technique [1,9,10]. When the center is fully operational, about 1300 patients will be treated every year using two treatment rooms with simple horizontal beam control and one with a gantry providing the opportunity for a 360° beam direction.

The next step will be to allow patients treated in the other particle therapy centers, firstly CNAO in Pavia, Italy, to also be documented in the database system. Using web-based access, referring physician and co-researchers within the ULICE project can access “their” data and provide additional information as well as follow-up data.

The system will offer the unique possibility to document specific clinical protocols or rather, studies, as summarized in Table 1.

**Table 1:** Studies being initiated at the Heidelberg Ion Therapy center (HIT)

**Study**	**Indication**	**Treatment concept**
CLEOPATRA	Primary glioblastoma	H1 vs. C12 Boost
CINDERELLA	Recurrent or progressive glioma	C12 vs. standard therapy
CHONDROSARCOMA	Skull base chondrosarcoma	H1 vs. C12
CHORDOMA	Skull base chordoma	H1 vs. C12
MARCIE	Atypical meningioma grad 3 and 4	C12 Boost
COSMIC	Salivary gland tumor	C12 Boost
ACCEPT	Adenoid cystic carcinoma	C12 Boost
PROMETHEUS	Hepatocellular carcinoma	C12
OSCAR	Inoperable osteosarcoma	H1 + C12
IPI	Prostate carcinoma	C12 Boost
PANDORA	Rectal carcinoma	C12
TPF	Head-neck tumor	C12 Boost
PHOENIX-01	Pancreatic cancer	C12
PINOCCHIO	Low-Grade meningioma	Photon vs. H1 vs. C12

### 3-Phase plan to attain a common database

It is still not unusual for clinical study documentation to be achieved with collections of paper-based case report forms (CRFs), excel sheets and local copies of medical images. It is not necessary to explain the disadvantages of such unstructured and distributed documentation. The main goal of our approach is to provide a central, web-based database and documentation system, with interfaces to the main existing information systems of the hospital for data import, to avoid double entries of patient and clinical data wherever possible.

To accomplish this goal, we designed a 3-phase plan. Phase 1 consists of an overall analysis of the patient workflow during a radiation therapy at the HIT; choosing a basic documentation system, which can be easily adapted and altered as necessary; and designing and implementing the basic modules for overall documentation.

The main part of the second phase is to connect the mandatory information systems of the hospital and the implementation of HL7 and DICOM interfaces for data import. The main input consists of overall treatment and follow-up data, physical data such as treatment plans, total dose and dose distribution as well as biological, molecular and pathological information. Security and data protection measures are implemented to fulfill legal requirements. Furthermore, the first specific clinical studies are designed and generated.

In phase 3, all existing clinical studies, particularly multicenter studies, are included in the database system. Therefore, the web-based access as an international platform for joint clinical research will be realized. In addition, the system will be used as a referring system. Partners desiring to use this new technology for their patients are able to request particle therapy and receive assistance with organizing complex treatment sequences.

Naturally, it is necessary to be able to follow the course of treatment at all times in order to provide optimal patient care [11]. Especially for external patients, knowledge of previous treatment is vital for the responsible oncologist in order to plan ion radiation. Therefore, all data for evaluating the case must be uploaded. Understandably, the referring physician himself is interested in the progress of treatment, while both parties will be awaiting the follow-up results.

## Results

After a period of eight months, we completed phase 1 in May of 2010 and are now intensively working on phase 2. We plan to start phase 3 in early 2012 and finish the project by the end of 2013. Electronic documentation was started in May 2011, and all patients are being documented. The following sections describe our results from the first two phases and the current progress.

### General principles and architecture

The basis of the documentation system is built with an open source PostgreSQL database with standard interfaces to the PACS world. It is based on the DICOM data model and can be dynamically extended with additional data structures. Interfaces allow the exchange and process of DICOM data as well as other information via HL7 messages.

A telemedicine record [12] functioning as an extension is added with the characteristic of an electronic patient record (EPR) and a professional DICOM viewer (Class IIb; according the European Medical Devices Directive). It allows the user to exchange, store, process, and visualize text data, all types of DICOM images and other multimedia data.

This general infrastructure was originally developed by the CHILI GmbH, a company specialized in radiology systems with whom we are privileged to maintain a strong cooperation and to whom we attribute the technical know-how and experience [13]. Based on our vision for the ULICE project, we have planned and implemented additional functionality and customized the general setting, thus creating a specialized study documentation system.

### Data presentation and storage

On the one hand, data can be imported to the system automatically via the mentioned standard DICOM and HL7 interfaces. On the other hand, the user can import it manually. This is done by entering single values into the interactive documentation modules or by web-upload of any multimedia documents through an interface, which has been implemented as a Java applet running in any Internet browser. Furthermore, a long-term archive (6 TB) is available to store and backup all DICOM, multimedia and documentation data.

The web-based graphical user interface is independent both from the running operation system (e.g. MAC OS, Linux, MS-Windows) and the used browser (e.g. Safari, Mozilla, Internet Explorer). Presentation and access to patient information is always patient-oriented. First, after logging in, the system shows the list of all patients included in that particular study, for whom the user is authorized. The data shown in the list can be configured for each study as needed. In addition, the list can be sorted and filtered individually.

**Figure 1:**
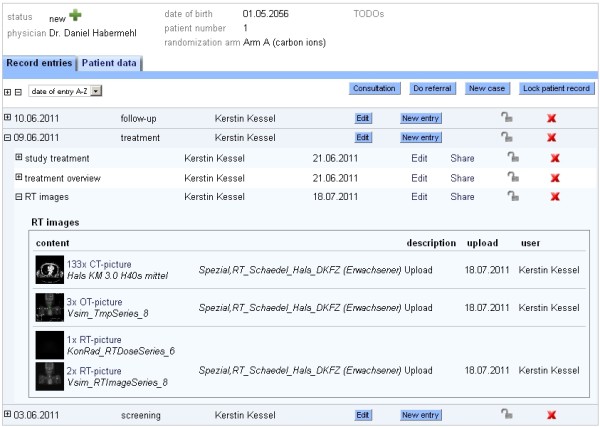
Screenshot showing a patient record with radiation information linked to the study documentation.

**Figure 2:**
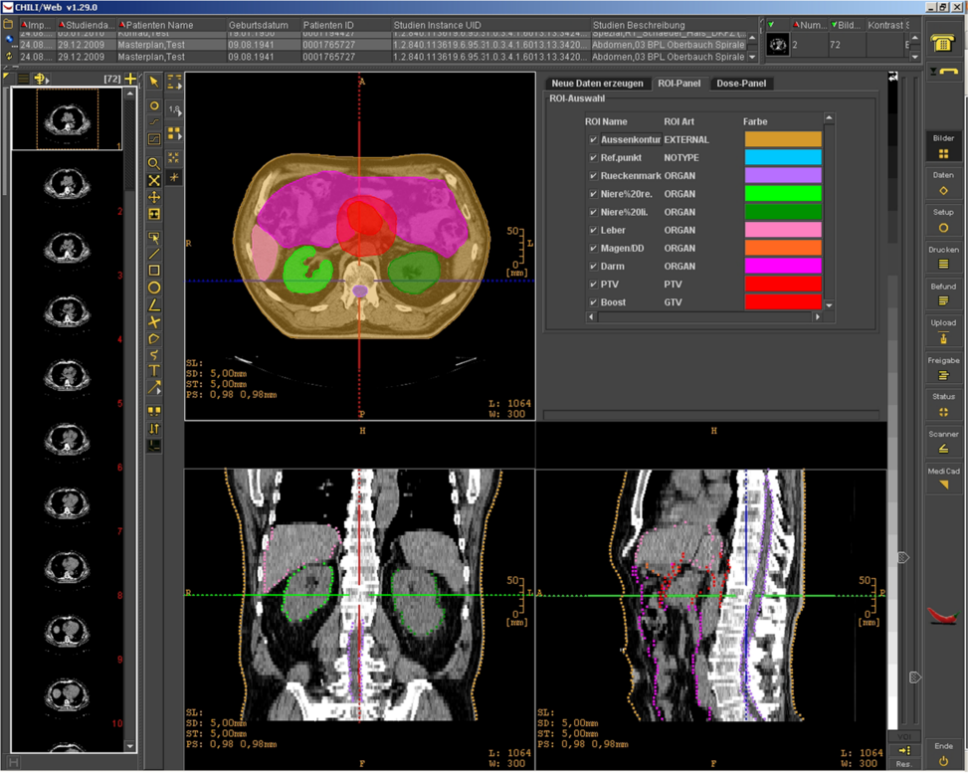
**Screenshot of our web-based DICOM-RT viewer showing the ROIs of a radiation plan in the transversal (top left), coronal (bottom left) and sagittal (bottom right) layers.** The corresponding colors are listed at the top right.

**Figure 3:**
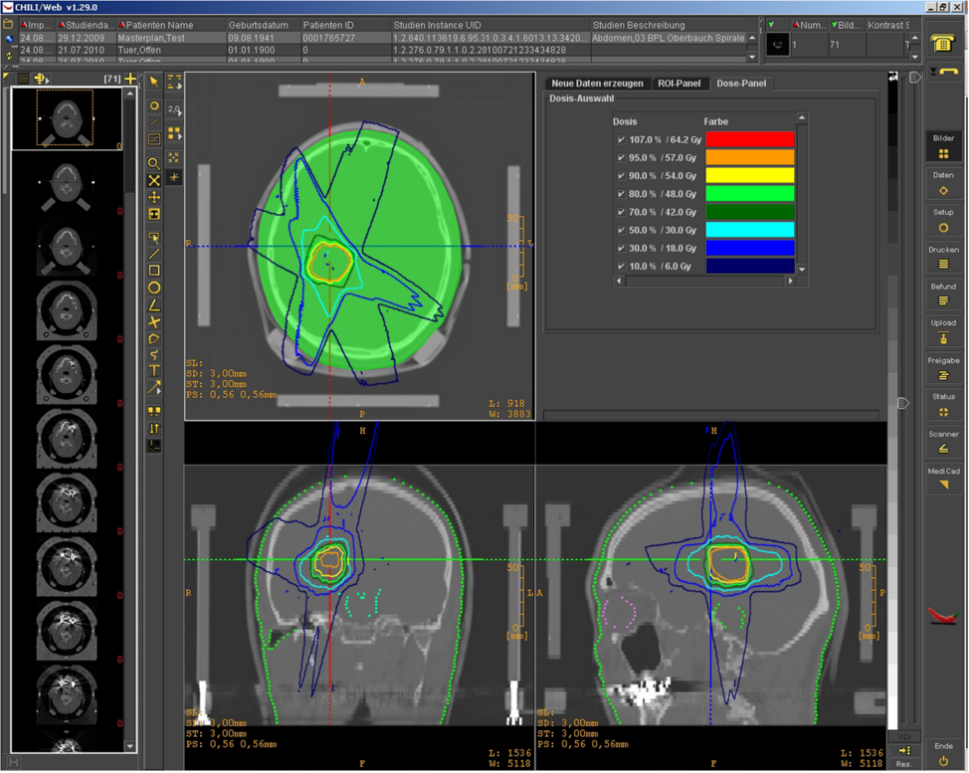
**Screenshot of our web-based DICOM-RT viewer showing the dose distribution of a radiation plan in the transversal (top left), coronal (bottom left) and sagittal (bottom right) layers.** The corresponding colors are listed at the top right.

After selecting one patient record, basic patient information is shown in the header as well as the created module entries for this patient. Figure 1 shows the view of a single patient with integrated radiation information. With a click on an imaging thumbnail, the DICOM-RT viewer is opened displaying the corresponding examination (see Figure 2 and Figure 3). This viewer is also web-based, can be executed from every workstation in the hospital, and will even be available for external users. Currently, we are developing the extension for visualizing the dose distribution and dose-volume histogram (DVH).

### Workflow integration

**Figure 4:**
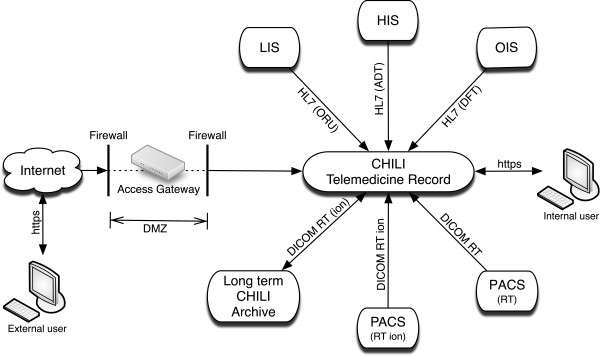
Connected systems and used protocols.

The system is connected with the Hospital Information System (HIS), the Laboratory Information System (LIS), the Picture Archiving and Communication Systems (PACS) and the Oncology Information System (OIS) in order to acquire automatic input via HL7 messages and DICOM (see Figure 4). Interfaces to the HIS provide the initial setting of patients with HL7-ADT messages. Laboratory findings such as blood results are automatically imported via HL7-ORU messages from the LIS, and interfaces to the PACS reveal radiation data by DICOM RT and DICOM RT ion (e.g. DICOM RT image/structure set/plan/dose/treatment record, CT and MR imaging). The OIS supplies further radiation information such as first and last day of irradiation, radiation method, and the applied particle-type over HL7-DFT messages. These messages are also used to trigger a DICOM Q/R (Query/Retrieve) on the different PACS to automatically import radiation data into the documentation system and map it to the corresponding patient (see Figure 1).

One external user is, for example, a referring physician possibly from a collaborating partner from abroad. He can upload all existing imaging information and detailed treatment data (e.g. surgery findings, blood results) from the patient’s previous treatments into the documentation system over the Internet. The physician at the HIT will review the case and decide if an indication for particle therapy is given and possibly a study inclusion. If the patient is accepted for an irradiation in Heidelberg, all further essential data is documented in the system. After the particle therapy, the referring physician continues the common documentation during the follow-up period for a complete treatment overview. As external PACS are not connected, there is always the possibility for manual upload for DICOM or any other multimedia data.

### Security concept

Since the system is used in a clinical environment, specific privacy and security mechanisms are required. The system uses the https protocol to exchange data between the central server and other systems (see Figure 4). This enables an encrypted data transfer.

Users need an account and password to access the system. A roles-and-rights concept has been established to configure access to each study of the system separately. So far, we created the main roles: physician (level 1, level 2), study nurse / case manager, physicist, simple user (e.g. student) and admin. Additionally, we can merge users into user groups, for example, all users from a specific institution. The roles of a user can change dynamically and are study-dependent, i.e. being a leading physician (level 1) for one study, and for another a standard user with only reading rights.

Furthermore, all data can be pseudonymized by a PID-Generator by the TMF (Technology, Methods, and Infrastructure for Networked Medical Research) [14], when it is imported into the system. The original patient information is then kept in a separate database. When a user has the right to view all data of a patient, the data is de-pseudonymized instantaneously - but only for the specific user. One user is typically the treating physician with full access to all data. Other users, e.g. researchers or external physicians, only have restricted access to pseudonymized data.

Communication with external users from participating study centers is realized by an intermediate application gateway in the demilitarized zone (DMZ) which receives https-requests, checks them and sends only valid requests to the server in the intranet of the hospital (see Figure 4). Additionally, client certificates are used to provide more host-to-host security and to verify authorized browsers of external project partners.

### Study and module design

In principle, each clinical study is designed and administered separately, consisting of several single modules. However, once a module is designed, it can be reused again. To this end, the documentation system has a graphical advanced administration tool, which includes a form generator for designing and adjusting specific modules for the individual clinical studies. It covers both the generation of the data structures and the corresponding graphical user interface. A major advantage is that this tool can be used by the local system administrators and does not need new developments by computer experts. Aside from that, a test system has been set up where new modules can be created, validated, and tested before they are used in the productive environment.

Very early, we decided not only to document patients treated within specific focused study protocols, but also patients not participating in clinical studies (non-study patients), meaning all patients ever treated with particle therapy, because, of course, not every patient fulfills the inclusion and exclusion criteria of a particular study. Thus, a default study is available where all patients are documented initially, until it is decided that they take part in a clinical study. In that case, a patient can be assigned to the study accordingly within the documentation system.

This can only be achieved by similar basic documentation for each type of patient (study or non-study) and led us to develop three different kinds of modules. Table 2 summarizes all existing modules. Basic modules are used for all patients and include vital patient information. The module for the treatment overview contains information such as diagnosis (ICD-O), TNM classification, tumor region and planned radiation therapy as well as previous oncological therapies. Radiation data, such as DICOM-RT, CTs and MRIs are stored in the RT images module; the corresponding precise details (e.g. dose information, number of fields, time and organs at risk) are mapped into the RT documentation module. The case management module is used for organizational data management, i.e. the health insurance, payment status, etc.

**Table 2:** Documentation modules divided into basic modules and modules for non-study and study patients

**Basic modules**	**Non-study modules**		**Study modules**
Basic patient data	Region module		Screening
Treatment overview	Head / neck		Inclusion / exclusion criteria
Case management	Brain / skull base		Pre-study treatment
RT images	Upper GI		Study treatment
RT documentation	Lower GI		Last examination
	Spine		Follow-up
	Pelvis		AE (Adverse Event)
	Extremities		SAE (Serious Adverse Event)
	Recurrence /metastasis		
	Death		

The study modules are tailored to fit the CLEOPATRA study.

For non-study patients, depending on the location of the tumor region, a specific region module must be used for documentation during screening, treatment and follow-up periods. These modules contain information that is again similar for all region types, for example karnofsky index, acquisition date of imaging, tumor diameter and response. However, corresponding symptoms and side-effects are documented individually. The recurrence / metastasis module documents the location and treatment method of the recurrence / metastasis; the death module the date and cause of death.

Furthermore, each clinical study has its own modules specifically designed to document the parameters that are required by the study protocol and / or CRF.

### Usability

Clinical documentation and analyses are crucial for an optimal patient care and medical research. However, this is not a particularly popular task. For this reason, we aim to replace manual input with automatic documentation wherever possible. Several features are implemented to support the documentation process and prevent double entries patient information. Certain modules are only selectable if predefined conditions are fulfilled. Some modules are only selectable once for each patient or depending on a previously created file entry. So-called listeners are implemented to fill data fields automatically, such as the time interval between surgery and the particular day or the age at study entry. Links to existing file entries within the documentation system help to switch between corresponding information. A patient-related link accessing the HIS from the documentation system makes it easy to search for additional patient information that is not part of the documentation system. Furthermore, a web-based ICD-O selector is implemented as an add-on feature for a standard documentation of diagnoses only allowing the search and insertion of valid encodings. We added a consultation feature for physicians to review findings or to obtain a second opinion. With the lock-option, a module-entry, a group of entries or even a whole case can be locked and thereafter not changed again by anyone. This is relevant for monitoring studies.

### Analyses

This task is still work in progress. Presently, data entries from the database can be exported as an Excel sheet. Thus, physicians can produce statistical reports for treatment-related questions within seconds - always up-to-date with the current status of information. To allow this, a query builder has been implemented, which supports the user to generate individual queries very intuitively. Additionally, these can be saved and reused any time for a continuous overview on the data. Case managers are using this functionality to monitor upcoming patient visits and to ensure complete and correct documentation.

## Discussion

The present manuscript describes the generation of a common European database and documentation system on particle therapy developed within the ULICE project. The goal is to summarize all biological, physical, and clinical data in one European system to generate a broad dataset on particle therapy. Hence, new treatment standards will be developed and tested on the basis of further clinical studies within ULICE. Each study can be designed and adjusted as needed within the system. By documentation of all patients ever treated with particle therapy, we can evaluate single studies prospectively and gain the possibility for overall large-scale, retrospective studies.

Different information systems such as the HIS, OIS and PACS have been connected successfully, allowing documentation and evaluation of different analyses.

All access to the documentation system takes place via the web. Thus, no software needs to be installed on clinical computers, as a web-browser is standard, and therefore the system can be immediately accessed everywhere on the Internet. The only prerequisite is an installed Java Runtime Environment (JRE). The web-based approach with its strong security measures allows the usage of the system for multicenter studies within the ULICE project and enables the essential patient referral functionality. Moreover, this will play a crucial role for the follow-up documentation. With the web-based access we can directly receive outcome reports by the physicians and even patient-reported-outcome reports according to the protocol, assessed in the vicinity of the patient’s home.

The usage of the documentation system is very simple and user-friendly as proven by first feedback from study nurses, case managers and physicians. The interactive documentation modules with their many features prevent wrong data input and guarantee data validation and verification.

In the environment of radiotherapy, it is essential that any DICOM RT data can be processed and visualized by all systems. Processing and display of RT data today is not yet a standard functionality of PACS or teleradiology systems. However, our system is able to exchange and store all kinds of DICOM RT data. The innovative, integrated and web-based DICOM viewer ensures examination of radiation plans from every single computer in the hospital. This enables physicians to quickly review images without having to go to a PACS or even a radiation therapy planning station.

Many others have already said there is no “one-size-fits-all” solution for web-based documentation of clinical studies or patient data per se [15]. The large number of requirements and circumstances for the system make an individual approach necessary. Our solution differs from other systems, which either only manage and organize patient treatment within a single department [16,17] or other numerous approaches only focused on electronically documenting a single clinical study [18-21]. We combine both. On the one hand, we developed a common platform that allows us to coordinate clinical studies in radiation oncology even across departments, and on the other hand, we linked it all to the mandatory information systems to manage a complete treatment overview with more detailed information that might be proven to be relevant in retrospect.

## Conclusion

The major benefit of this system lies in the fact that imaging information, i.e. RT, CT and MRI data is directly linked to the rest of the study, or rather treatment documentation (see Figure 1) and can be simply and quickly accessed with standard web-browsers. This, in turn, simplifies the process of conducting multicenter studies distributed all over Europe. It gives us the opportunity to extend the analysis functionality to a more complex level. With the main aim to reduce the effort for future clinical studies, we are planning a separate functionality for prospective and retrospective data analyses. It will not only be able to answer simple statistical questions, but also considering imaging information. MR imaging and dose distribution are to be compared before and after treatment and thus reveal their direct correlation with clinical endpoints (e.g. overall survival, disease-free survival, recurrence location).

In conclusion, the documentation system of today simplifies the research work, ensures a better quality of study analyses, and ultimately improves patient treatment concepts and supports the evaluation of the role and effectiveness of particle therapy.

## Competing interests

There is no conflict of interest to report for this article.

## Authors’ contributions

KAK, NB, CB, DO, RB, UE, RO, PF, RP, JD and SEC designed the common particle database. KAK, NB, CB and UE developed the system. KAK drafted and wrote the manuscript. DH, DO, JD and SEC were responsible for patient treatment and care. NB, CB, DH, UE, MD and SEC reviewed/revised the manuscript. All authors read and approved the final manuscript.
